# Relationship between Active Aging and Quality of Life in Middle-Aged and Older Koreans: Analysis of the 2013–2018 KNHANES

**DOI:** 10.3390/healthcare9020240

**Published:** 2021-02-23

**Authors:** MiJung Eum, HyungSeon Kim

**Affiliations:** 1 Department of Nursing Science, Kyungbuk College, 77 Daehak-ro, Yeongju-si 36133, Gyeongbuk, Korea; emj44@hanmail.net; 2 Department of Nursing, Bucheon University, 56 Sosa-ro, Bucheon-si 14774, Gyeonggi-do, Korea

**Keywords:** health-related quality of life, active aging, middle aged, aged

## Abstract

With the increase in the aging population worldwide, social interest in having a vibrant and valuable old age has been increasing with changes in the perspectives on old age. This study aimed to determine the relationship between active aging and health-related quality of life (HRQOL) in middle-aged and older Korean using national data. The subjects were 14,117 adults aged ≥55 years. HRQOL was evaluated using the EuroQol–5 Dimension (EQ-5D) questionnaire, and active aging was defined based on the health factors, participation factors, and security factors. The average EQ-5D score was 91.04 ± 0.143. Hierarchical multiple regression analysis sequentially inputting the health, participation, and security factors showed that health factors had the strongest influence on HRQOL (F = 216.656, *p* < 0.001). In the final model, which included all variables, activity limit (B = −10.477, *p* < 0.001) and subjective health status (B = −7.282, *p* < 0.001) were closely related to the HRQOL. In addition, economic activity, income level, home ownership, private health insurance, and unmet healthcare needs were associated with HRQOL. The R^2^ of the model was 38.2%. To improve the HRQOL of middle-aged and older people, it is necessary to consider active aging factors. Furthermore, follow-up studies using various indicators reflecting active aging should be conducted.

## 1. Introduction

According to the United Nations Population Fund, the world population is aging rapidly, with people aged ≥60 years accounting for 12.3% of the world’s population, and this number is projected to increase to nearly 22% by 2050 [1]. South Korea became an aged society in 2019, with 14.9% of the population aged ≥65 years, and the proportion is expected to increase to 46.5% by 2067 [2]. In particular, as Korea took only 18 years to enter the aged society due to a continuous decline in childbirth and prolonged life expectancy, Korea received attention as the country with the fastest pace of aging among the Organization for Economic Co-operation and Development (OECD) countries [3]. Therefore, compared with other developed countries that have prepared for an aging society, Korea faces various problems related to aging, such as economic difficulties experienced in old age, increasing physical and mental chronic diseases, death of a spouse, loss of role, and social isolation. All these situations made it necessary for Korea to prepare for the problems urgently [4].

As social interest in the valuable life of old age has increased due to longer life expectancy, negative perceptions about old age have changed and people of old age have been redefined as positive and active members of the society [5]. The concept of ‘active aging’ has been acknowledged by international organizations due to a new paradigm shift in aging populations [6]. The World Health Organization (WHO) defines active aging as the process of optimizing opportunities for health, participation, and security to enhance the quality of life as people age [7]. Attention should be focused on the factors related to the life of the aging population, because interest in improving the older adults’ quality of life gradually increases in accordance with the national income, living standards, and the prevalence of chronic diseases; in particular, the quality of life of older adults is closely related to the quality of life at the national level. 

The WHO suggested that health, participation, and security factors are components of active aging. They also indicated that these three factors are closely related to each other, health and security factors activate participation factors, and health and security factors can be enhanced by participation factors [7].

According to previous studies, the quality of life of older adults is affected by general characteristics such as age and education level [8,9] and lifestyle characteristics such as smoking, drinking, and sleep [10,11,12]. Moreover, quality of life is closely related to physical and mental status [13,14,15]; it is helpful for older adults to frequently engage in leisure activities to maintain quality of life [16], and the employment status of older adults has a greater relevance to quality of life compared with younger adults [17,18]. 

Active aging has a positive effect on the quality of life [19], and is understood in relation to physical, cognitive, psychological and social factors, but these co-exist in complex combinations [20]. Although there is a significant relationship between active aging and quality of life, most countries do not offer the generalized conditions of a proper life quality for their older population [21]. Hence, a strategy to provide these conditions should be established. Although interest in investigating the components of active aging has gradually increased in recent years with the increase in the older population, there is insufficient evidence showing the sub-factors that constitute active aging; studies tend to focus mainly on the aging population in Western countries, and selectively apply or analyze those parts that constitute active aging. In addition, studies evaluating the relationship between active aging and quality of life are limited in quantity.

Therefore, this study aimed to define the components of active aging and identify the level of health-related quality of life (HRQOL) using a large national sample of people aged ≥55 years from the 2013–2018 Korean National Health and Nutrition Examination Survey (KNHANES). We also aimed to identify active aging factors that affect the HRQOL of middle-aged and older adults.

## 2. Materials and Methods

### 2.1. Study Design

This was a secondary analysis using the 2013–2018 KNHANES as a cross-sectional study to identify the active aging factors that affect the HRQOL in middle-aged and older Koreans.

### 2.2. Research Subjects

This study used the 6-year data, which included a combination of the sixth (2013–2015) and seventh (2016–2018) data, from the KNHANES [22]. The KNHANES is a national surveillance system that has been assessing the health and nutritional status of Koreans since 1998. It is conducted by the Korea Centers for Disease Control and Prevention (KCDC) every 3 years using nationally representative samples of the civilian non-institutionalized Korean population. The sampling plan followed a multi-stage clustered probability design. Health interviews and health examination surveys were conducted. Of the 47,217 survey participants, only 14,117 were included in this study. Participants aged <55 years (*n* = 30,765) and those with missing survey data (*n* = 2335) were excluded.

### 2.3. Definition of Variables

#### 2.3.1. Quality of Life

The EuroQol–5 Dimension (EQ-5D) questionnaire developed by the Euro-Qol Group was used in the KNHANES to measure HRQOL. The EQ-5D questionnaire consists of five items: mobility, self-care, usual activities, pain/discomfort, and anxiety/depression. Each dimension is represented by a single item with three levels of responses: no problem, some problems, and extreme problems. Each item in the questionnaire is rated as follows: 1 point, no problem or no inconvenience; 2 points, some problems or inconveniences; and 3 points, extreme problems or unable to do anything. The KNHANES used the Korean version of the EQ-5D questionnaire which was already developed and proved its validity and reliability to investigate HRQOL [23,24], and provided the EQ-5D index score. In this study, the value obtained by converting the EQ-5D index score to a perfect score of 100 was used. To check the distribution of the different items in the EQ-5D questionnaire, the response ‘no problem or no inconvenience’ was changed to ‘no problem status’, while ‘some problems or inconveniences’ or ‘extreme problem or unable to do anything’ was changed to ‘problem status’.

#### 2.3.2. Active Aging

In this study, five health factors, two participation factors, and four security factors were selected as components of active aging based on the definition of the WHO [7] and previous studies [25].

##### Health Factor

Health factors included medical physical health status, functional physical health status, and mental health status. Medical physical health status was defined as the number of chronic diseases (0, 1, or ≥2), while functional physical health status was defined as limitation in physical activities (yes or no). The number of chronic diseases referred to the presence of at least one type of cancer, circulatory disease, musculoskeletal disease, endocrine disease, respiratory disease, and digestive system disease. Mental health status was defined based on the level of stress (much or little), subjective health status (good, moderate, or bad), and status of depression (yes or no).

##### Participation Factor

Participation factors were defined based on the status of economic activity (yes or no) and household type (1st generation, 2nd generation, or 3rd generation).

##### Security Factor

Security factors were defined based on the income level (1st quartile, 2nd quartile, 3rd quartile, or 4th quartile), type of home ownership (yes or no), status of private health insurance (yes or no), and status of unmet healthcare needs (yes or no).

#### 2.3.3. General Characteristics

Demographic characteristics included sex (men or women), age (55–64 years or ≥ 65 years), education level (elementary school, middle school, high school, or college or higher), and the presence of a spouse (yes or no). The health behavior characteristics included smoking (yes or no), alcohol use (yes or no), physical activity (yes or no), and average sleep duration (<6, 6–8, or ≥9 h).

### 2.4. Statistical Analysis

Since the KNHANES data were obtained through multistage clustered sampling, all data were analyzed after designing a composite sample with a weighted frequency. The general characteristics of the study subjects and the level of HRQOL were presented as frequency, percentage, mean, and standard error were expressed using descriptive statistics. The relationship between the factors of active aging and HRQOL was analyzed using a *t*-test and analysis of variance. Hierarchical multiple regression analysis was performed to identify the active aging factors that affect HRQOL. In Model I, the general characteristics of the subjects, such as sex, age, education level, presence of a spouse, smoking, alcohol use, physical activity, and average sleeping duration were analyzed. In Model II, among the components of active aging, health factors such as number of chronic diseases, limitations in physical activities, the level of stress, subjective health status, and status of depression variables were added to Model I. In Model III, among the components of active aging, participation factors such as status of economic activity and household type were added to Model II. Lastly, in Model IV, security factors such as income level, type of home ownership, status of private health insurance, and status of unmet healthcare needs were added to Model III. All statistical analyses were performed using SPSS Statistics version 23 (IBM Corp., Armonk, NY, USA), and the statistical significance level was set to a two-sided test at *p* < 0.05.

### 2.5. Ethical Considerations

All individuals who participated in the KNHANES conducted by the KCDC provided informed consent, and the participants’ names were anonymized. This study was approved by the Research Ethics Review Committee (P01-202101-21-001).

## 3. Results

### 3.1. General Characteristics of the Subjects

The demographic and health behavior characteristics of the participants are presented in Table 1. Among the included participants, 45.8% were men and 54.2% were women. In terms of age, 51.8% participants were aged 55–64 years, while 48.2% were over 65 years. With regard to education level, 42.5% were elementary school graduates. 74.7% participants had a spouse and 25.3% participants had no spouse. Meanwhile, 14.3% were smokers, 43.9% were alcohol drinkers, and 37.2% participants performed physical activities. In terms of average sleep duration, 18.1% participants slept less than 6 h, while 9.2% participants slept more than 9 h.

### 3.2. Health-Related Quality of Life

Table 2 shows the participants’ HRQOL. The average EQ-5D score was 91.04 ± 0.143. The proportions of respondents with ‘problem status’ were 31.9% for pain/discomfort, 25.7% for mobility, 14.5% for usual activities, 13.5% for anxiety/depression, and 7.2% for self-care. 

### 3.3. Health-Related Quality of Life According to the Components of Active Aging

Table 3 shows the HRQOL according to the components of active aging. The components of active aging were classified into health, participation, and security factors. Among the health factors, older adults with two or more chronic diseases had a lower quality of life score than those without chronic disease (*p* < 0.001). In terms of functional and physical health, older adults with activity limit had a lower quality of life score than those without activity limit (*p* < 0.001). With regard to mental health, older adults with stress, poor subjective health, and depression had a lower quality of life score (*p* < 0.001). In terms of participation factors, older adults who engaged in economic activities and belonged in a second-generation household had a higher quality of life score (*p* < 0.001). In terms of security factors, first quartile income earners, those who did not own a house, those who were not enrolled in private health insurance, and those with unmet healthcare needs had a lower quality of life score (*p* < 0.001).

### 3.4. Active Aging Factors Affecting Health-Related Quality of Life

Table 4 shows the active aging factors that affect HRQOL. Multiple regression analysis was conducted by hierarchically inputting the general characteristic factors and components of active aging, such as health factors, participation factors, and security factors. As a result, the R^2^ of demographic characteristics in Model I was 14.0% (F = 207.187, *p* < 0.001). HRQOL was higher in men than in women and in older adults who had a spouse than those who didn’t have a spouse. The HRQOL was low in participants aged over 65 years, with a low level of education, who were smokers, and with a sleep duration of less than 6 h or more than 9 h compared with those with a sleep duration of 6–8 h. 

The R^2^ of Model II was 36.1% (F = 277.398, *p* < 0.01), which increased by 22.1% *p* after introducing the health factors. HRQOL was lower in participants with two or more chronic diseases, high level of stress, and depression. Moreover, those with activity limits (B = −11.367, *p* < 0.001) and those with poor subjective health (B = −7.888, *p* < 0.001) had a significantly lower HRQOL. 

The R^2^ of Model III was 36.4% (F = 271.572, *p* < 0.001), which increased by 0.3%*p* after introducing the participation factors. The HRQOL of older adults who engaged in economic activities was higher than that of older adults who did not engage in economic activities (B = 1.803, *p* < 0.001). 

The R^2^ of Model IV was 38.2% (F = 216.656, *p* < 0.001), which increased by 1.8%*p* after introducing the security factors. The HRQOL was lower in older adults with the lowest income level (1st quartile) than in those with the highest income level (4th quartile); HRQOL was higher in older adults who owned a house than in those who did not own a house. In addition, the HRQOL was higher in those who had private health insurance than in those who did not have private health insurance (B = 1.075, *p* < 0.001). Those who did not meet healthcare needs showed remarkably lower quality of life compared with those who met healthcare needs (B = −5.518, *p* < 0.001). 

As a result of comparing the absolute values among variables in Model IV, in which all variables were included, activity limit (B = −10.477, *p* < 0.001) and subjective health status (B = −7.282, *p* < 0.001) had the strongest influence on the HRQOL of middle-aged and older people.

## 4. Discussion

The concept of active aging has a limitation. The definitions presented by the international organizations are inconsistent and lack specific indicators and measurements. However, in many countries, it has had a considerable impact on the change of perspectives on old age and the establishment of new strategies [26,27,28]. In defining the concept of active aging, while the WHO prioritizes the social aspects of health and quality of life, the European Union (EU) and the OECD emphasize the capacity of older adults to contribute to the economy and society with a greater focus on increasing economic productivity during old age [29]. Older people or their families cannot usually solve the problems that they are facing themselves. Hence, these problems must be viewed from a comprehensive perspective of improving the quality of life, not simply expanding economic productivity.

The main result of this study, which was conducted to confirm the relationship between active aging factors and HRQOL in Korean middle-aged and older adults, is that health factors, participation factors, and security factors defined as components of active aging are related to HRQOL. Among them, activity limit and subjective health status which are health factors were closely related with HRQOL. 

Older adults are more likely to have difficulty in physical activity as they age. Even in a study of people over 50 years old, those with physical function restrictions had poor physical and mental health conditions and HRQOL [30]. Physical function restrictions can be disadvantageous to access to the social environment and support. To improve the quality of life of older adults, it is necessary to consider the activities that minimize physical function restrictions to determine the factors that need priority. Subjective health status is an important measure for assessing HRQOL and is directly related to quality of life [31]. Subjective health status was a factor affecting quality of life, even for older adults with chronic diseases [32]. Older adults with good physical function showed better subjective health status [33], and those who had more social activities perceived their status more positively [34]. As life expectancy increases, the prevalence of chronic diseases among older adults is also increasing, and chronic diseases are the factors associated with low HRQOL [35]. The most frequent degenerative diseases leading to poor quality of life include cancer, hypertension, osteoporosis, and diabetes [36]. Among them, older adults with arthritis have limited social participation and physical activities compared with those without arthritis, leading to a decrease in quality of life [15]. This finding implies that chronic diseases that worsen physical function restrictions can lower the quality of life. According to the ‘National Survey of Older Persons’, the average number of chronic diseases among older Korean adults increased from 1.5 cases in 2008 to 2.7 cases in 2017, and the proportion of older adults with three or more chronic diseases increased significantly from 30.7% to 51.0%. Approximately 8% of older adults reported that their daily activities were limited [37]. Therefore, early detection and management of chronic diseases that can worsen physical function restrictions in older adults should be recognized as an important factor for improving the HRQOL of the elderly and the establishment of national strategies is desperately needed.

In this study, the HRQOL of older adults who engaged in economic activities was higher than HRQOL of those who did not engage in economic activities (B = 1.732, *p* < 0.001). Another study using panel data also reported that people engaged in paid work during old age had a high quality of life [38]. However, the quality of life of older adults who engaged in physical labor was lower than that of office workers and was more affected by health factors than that of office workers [39]. Korea has a higher employment rate for older adults than European Union (EU) countries [40], and while the employment rate of the 65- to 69-year-old age group in Korea is higher than that of the OECD average, most of the older adults in Korea finish their core careers in thier early 50s and tend to obtain low-quality and unstable jobs [3]. Employment in old age is an important factor affecting quality of life [38,41]. Participation refers to the labor market, employment, education, health, and social policies and programs that support their full participation in the socioeconomic, cultural, and spiritual activities; according to their basic human rights, capacities, needs, and preferences, people will continue to make a productive contribution to society in both paid and unpaid activities as they age [7]. For the older adults’ economic activities to lead to active aging, various decent jobs must be provided by society. 

According to the security meaning of the WHO, when policies and programs address the social, financial, and physical security needs and rights of people as they age, older people are ensured of protection, dignity, and care in the event that they are no longer able to support and protect themselves [7]. We examined the social, financial, and health aspects of individuals and their effects on HRQOL. The HRQOL was low in the group with the lowest income level and with unmet healthcare needs. By contrast, HRQOL was high in the group that owned a house and with private health insurance. It was confirmed not only in this study but also in previous studies [42,43] that high income level and home ownership are obvious factors that positively influence the quality of life of older people. 

Unmet healthcare needs are a condition in which medical needs are not met [44]. These are factors that affect disease prognosis in terms of failure to detect disease early, increase in disease severity, and increase in the likelihood of complications [45]; this study also confirmed that unmet healthcare needs have a great influence on HRQOL (B = −5.518, *p* < 0.001). Private health insurance, which can also affect HRQOL, is a factor affecting the experience of unmet healthcare needs. In particular, it can greatly affect those who have experienced unmet healthcare needs for economic reasons [46], and older people with underinsurance are more likely to have the experience of unmet healthcare needs than those with adequate insurance [47,48]. In terms of the security factors of active aging, national policy efforts such as reducing the rate of unmet healthcare needs, as a significant security factor affecting the quality of life identified in this study, are needed. As we age, security needs and rights should be considered to improve quality.

Due to the rapid aging of the population, the average age of workers in the labor market is predicted to increase. The results of this study imply that it is necessary to understand the three factors constituting active aging and to approach them in multifaceted ways to improve the quality of life of people aged over 55 years.

The strength of this study is that it is highly representative as it uses large-scale data surveyed nationally and is a weighted composite sample analysis. However, this study has several limitations. First, considering that the study included middle-aged and older adults from Korea, the results are not necessarily generalizable to other population groups. Second, the possibility of under-reporting or over-reporting cannot be ruled out because the analysis results were based on the participant’s response to the questionnaire survey. Third, the variables used in the analysis of the three factors suggested as active aging factors were about partial aspects of the entire active aging components. For example, since the data on care activities, which is a representative index of the participation factors, were not obtained in the KNHANES, the type of household was used as a proxy indicator. Fourth, this was a cross-sectional study; hence, the causal relationship between active aging factors and HRQOL could not be inferred. Nevertheless, the finding of this study that active aging factors are closely related to the HRQOL of elderly individuals can be used as a basis for further studies. Based on the results of this study, we suggest an additional study to derive the relationship between HRQOL and various indicators that reflect active aging factors.

## 5. Conclusions

This study showed meaningful results regarding the relationship between active aging factors and HRQOL in middle-aged and older Koreans. The health factors, participation factors, and security factors that constitute active aging were related to HRQOL. In particular, among the health factors, subjective health status and activity limit were closely related to HRQOL. Therefore, a multifaceted approach including each factor of active aging is necessary to improve the quality of life of middle-aged and older adults.

## Figures and Tables

**Table 1:** General characteristics of subjects.

Variables	Category	N	%
Sex	Men	6097	45.8
	Women	8020	54.2
Age (years)	55–64	6168	51.8
	≥65	7949	48.2
Education level	Elementary	6486	42.5
	Middle school	2454	17.8
	High school	3213	24.4
	College or higher	1964	15.3
Spouse	Yes	10,402	74.7
	No	3715	25.3
Smoking	Yes	1804	14.3
	No	12,313	85.7
Alcohol use	Yes	5828	43.9
	No	8289	56.1
Physical activity	Yes	5074	37.2
	No	9043	62.8
Sleep duration(h)	<6	2616	18.1
	6–8	10,094	72.7
	≥9	1407	9.2

**Table 2:** Health-related quality of life.

Variables	Category	N(%)
EQ-5D		91.04 ± 0.143 *
Mobility problem	Yes	3967 (25.7)
	No	10,150 (74.3)
Self-care problem	Yes	1130 (7.2)
	No	12,987 (92.8)
Usual activities problem	Yes	2243 (14.5)
	No	11,874 (85.5)
Pain/Discomfort problem	Yes	4681 (31.9)
	No	9436 (68.1)
Anxiety/Depression problem	Yes	2033 (13.5)
	No	12,084 (86.5)

* EuroQol–5 Dimension (EQ-5D) score; Mean ± SE (standard error).

**Table 3:** Health-related quality of life according to active aging.

Variables	Category	EQ-5D *	F	*p*-Value
**Health factors**	**Number of chronic diseases**			
	0	95.12 ^c^ ± 0.155	622.005	<0.001
	1	92.26 ^b^ ± 0.160		
	≥2	86.83 ^a^ ± 0.212		
	**Limitation in physical activities**			
	Yes	75.38 ± 0.437	1893.263	<0.001
	No	93.47 ± 0.097		
	**Level of stress**			
	Much	84.98 ± 0.319	630.001	<0.001
	Little	92.54 ± 0.140		
	**Subjective health status**			
	Good	97.01 ^c^ ± 0.099	1813.331	<0.001
	Bad	80.50 ^a^ ± 0.273		
	Moderate	93.63 ^b^ ± 0.142		
	**Depression**			
	Yes	81.91 ± 0.517	350.670	<0.001
	No	91.60 ± 0.141		
**Participation factors**	**Economic activity **			
	Yes	94.26 ± 0.109	661.200	<0.001
	No	87.93 ± 0.246		
	**Household type**			
	1st generation	90.25 ^a^ ± 0.223	48.118	<0.001
	2nd generation	92.36 ^b^ ± 0.131		
	3rd generation	90.30 ^a^ ± 0.303		
**Security factors**	**Income level**			
	1st quartile	85.37 ^a^ ± 0.251	631.341	<0.001
	2nd quartile	91.59 ^b^ ± 0.151		
	3rd quartile	93.93 ^c^ ± 0.107		
	4th quartile	95.37 ^d^ ± 0.090		
	**Home ownership**			
	Yes	92.09 ± 0.157	155.936	<0.001
	No	87.78 ± 0.305		
	**Private health insurance**			
	Yes	93.64 ± 0.101	646.294	<0.001
	No	86.75 ± 0.261		
	**Unmet healthcare needs**			
	Yes	80.88 ± 0.321	1569.184	<0.001
	No	92.29 ± 0.129		

* Values are Mean ± SE (standard error); Bonferroni correction: a < b < c < d.

**Table 4:** Active aging factors affecting health-related quality of life.

Variables	Category	Model I *			Model II **			Model III ***			Model IV ****		
		B	SE	*p*-Value	B	SE	*p*-Value	B	SE	*p*-Value	B	SE	*p*-Value
**Sex** (ref. Women)	Men	1.486	00.332	<0.001	0.809	0.148	<0.001	0.487	0.153	0.002	0.367	0.172	0.038
**Age(years)** (ref. 55–64)	≥65	−3.557	0.203	<0.001	−2.666	0.220	<0.001	−2.185	0.235	<0.001	−1.763	0.212	<0.001
**Education** (ref. College or higher)	Elementary	−5.512	0.281	<0.001	−2.573	0.253	<0.001	−2.782	0.258	<0.001	−2.167	0.270	<0.001
	Middle school	−2.066	0.313	<0.001	−0.162	0.253	0.525	−0.361	0.257	0.165	−0.207	0.281	0.465
	High school	−0.836	0.190	<0.001	0.099	0.145	0.500	0.032	0.148	0.831	0.104	0.155	0.506
**Spouse** (ref. No)	Yes	3.749	0.272	<0.001	2.760	0.248	<0.001	2.768	0.248	<0.001	1.887	0.256	<0.001
**Smoking** (ref. No)	Yes	−0.886	0.301	0.005	−0.234	0.252	0.359	−0.215	0.252	0.398	0.099	0.234	0.674
**Alcohol use** (ref. No)	Yes	1.747	0.159	<0.001	0.613	0.148	<0.001	0.510	0.150	0.001	0.441	0.149	0.005
**Physical activity** (ref. No)	Yes	1.941	0.196	<0.001	1.104	0.185	<0.001	1.094	0.188	<0.001	1.088	0.196	<0.001
**Sleep duration(h)** (ref. 6–8 h)	<6	−2.607	0.234	<0.001	−1.562	0.214	<0.001	−1.513	0.202	<0.001	−1.236	0.216	<0.001
	≥9	−3.070	0.342	<0.001	−1.492	0.298	<0.001	−1.440	0.297	<0.001	−1.293	0.285	<0.001
**Number of chronic diseases** (ref. 0)	≥2				−1.595	0.198	<0.001	−1.490	0.201	<0.001	−1.572	0.194	<0.001
	1				−0.110	0.152	0.471	−0.058	0.154	0.709	−0.108	0.163	0.512
**Limitation in physical activities** (ref. No)	Yes				−11.367	0.421	<0.001	−11.140	0.411	<0.001	−10.477	0.404	<0.001
**Level of stress** (ref. Little)	Much				−3.494	0.239	<0.001	−3.635	0.239	<0.001	−3.185	0.240	<0.001
**Subjective health status** (ref. Moderate)	Good				1.414	0.131	<0.001	1.356	0.130	<0.001	1.206	0.135	<0.001
	Bad				−7.888	0.214	<0.001	−7.772	0.211	<0.001	−7.282	0.217	<0.001
**Depression** (ref. No)	Yes				−2.959	0.375	<0.001	−2.780	0.376	<0.001	−2.632	0.378	<0.001
**Economic activity** (ref. No)	Yes							1.803	0.189	<0.001	1.731	0.190	<0.001
**Household type** (ref. 3rd generation)	1st generation							−0.661	0.306	0.035	-.0.072	0.295	0.808
	2nd generation							−0.884	0.287	0.003	−0.553	0.289	0.062
**Income level** (ref. 4th Quartile)	1st Quartile										−1.020	0.290	<0.001
	2nd Quartile										0.176	0.224	0.438
	3rd Quartile										0.214	0.120	0.080
**Home ownership** (ref. No)	Yes										1.132	0.238	<0.001
**Private health insurance** (ref. No)	Yes										1.075	0.208	<0.001
**Unmet healthcare needs** (ref. No)	Yes										−5.518	0.196	<0.001
***R*^2^**		0.140			0.361			0.364			0.382		
**Δ*R*^2^**					0.221			0.003			0.018		
***F***		207.187 (*p* < 0.001)			277.398 (*p* < 0.001)			271.572 (*p* < 0.001)			216.656 (*p* < 0.001)		

* Adjusted for sex, age, education, Spouse, smoking, Alcohol use, Physical activity, Sleep duration. ** Adjusted for Model I + Number of chronic diseases, Limitation in physical activities, Level of stress, Subjective health status, Depression. *** Adjusted for Model II + Economic activity, Household type. **** Adjusted for Model III + Income level, Home ownership, Private health insurance, Unmet healthcare needs.

## Data Availability

Restrictions apply to the availability of these data. Data were obtained from KCDC and are available from https://knhanes.cdc.go.kr/knhanes/main.do.
